# Methodology for Preoperative Planning of Bone Deformities Using Three-dimensional Modeling Software

**DOI:** 10.1055/s-0044-1779700

**Published:** 2024-03-21

**Authors:** Cláudio Wanderley Luz Saab Filho, Mariana Demétrio de Sousa Pontes, Carlos Henrique Ramos, Luiz Antonio Munhoz da Cunha

**Affiliations:** 1 Serviço de Ortopedia e Traumatologia, Hospital Pequeno Príncipe, Curitiba, PR, Brasil; 2 Serviço de Ortopedia e Traumatologia, Hospital de Clínicas da Universidade Federal do Paraná, Curitiba, PR, Brasil

**Keywords:** printing, three-dimensional, orthopedic, preoperative care

## Abstract

Rapid prototyping technology, known as three-dimensional (3D) printing, and its use in the medical field are advancing. Studies on severe bone deformity treatment with 3D printing showed benefits in postoperative outcomes thanks to this technology. Even so, preoperative planning guidance for surgeons is lacking. This technical note describes a practical step-by-step guide to help surgeons use this technology to optimize the therapeutic plan with free license software and an intuitive interface. This study aims to organize the 3D modeling process using a preoperative computed tomography (CT) scan. This technology allows a deeper understanding of the case and its particularities, such as the direction, planes, and dimensions of the deformity. Planning considering these topics may reduce the surgical time and result in better functional outcomes by understanding the deformity and how to correct it. Associating planning via software with 3D printing can further enhance this therapeutic method.

## Introduction

Rapid prototyping technology, known as three-dimensional (3D) printing, and its use in the medical field are advancing. 1 The technology has been improved and applied in different ways. Orthopedics has been using this technology for education, better disease or deformity understanding, implant customization, preparation of custom-made orthoses, and preoperative planning. 1 2 3 4

Studies on severe bone deformity treatment with 3D printing showed benefits in postoperative outcomes thanks to this technology. 5 However, the literature providing surgical guidelines for preoperative planning using rapid prototyping remains scarce.

Since there is a number of software for 3D modeling 6 7 with a range of apparently complex learning curves at first glance, this technical note aims to describe a practical step-by-step guide to help orthopedic surgeons use this technology to optimize the therapeutic plan with free license software and an intuitive interface.

## Material Description and Technique

The Research Ethics Committee from our hospital approved this study under number (CAAE: 48296321.8.0000.0097).

This study aims to organize the software-based 3D modeling process using the preoperative computed tomography (CT) scan of the leg of a patient with Hajdu-Cheney Syndrome and a "serpentine" fibula deformity. The test occurred in the hospital's multislice CT scan, GE Healthcare Revolution, 64 channels (GE Healthcare, Chicago, IL, United States).

InVesalius software (Renato Archer Information Technology Center, Brazil) version 3.1.1 reconstructed the images, while modeling and editing used the Autodesk Meshmixer software (Autodesk Inc., San Rafael, CA, United States) version 3.5.474.

## Image Reconstruction

Meshmixer planning requires two files in stereolithography (STL) format: one of the affected bones and the other of the same bones without deformity for comparison.

First, the patient's CT image is downloaded directly from the viewing system available at the imaging service/hospital. The file must be in digital imaging and communications in medicine (DICOM) format for import into the InVesalius software. The selection of area of interest for STL conversion occurs as described by Pontes et al. 8

The comparison STL file may be the contralateral CT image in cases of unilateral deformities or CT images from other patients of a similar age group.

## Surgical Planning: Edition and 3D Modeling

Start the Meshmixer software, select the “import” option, and choose the deformity SLT file to load the 3D image. Initially, it is critical to know some commands required to proceed with modeling.

A right mouse click can rotate the model. The mouse scroll button allows you to zoom in or out of the model and click it to navigate in the horizontal and vertical planes.

The interface has two main bars, i.e., the top and the left side. The left sidebar presents the main editing tools, i.e., “Import”, “Meshmix”, “Select”, “Sculpt”, “Stamp”, “Edit, “Analysis”, “Shaders”, “Export”, and “Print”. For osteotomy planning, the main initial functions are ''Edit" to section and mobilize the fragments and “Shaders” for color changes to facilitate visualization.

The top bar of the software has the commands “File”, “Action”, “View”, “Help”, and “Feedback”. Clicking on “View”, select the option “Show objects browser” to facilitate editing in the following steps. When clicking for the first time, you will notice an object browser (“Object Browser”), then with a single object.

**Fig. 1:**
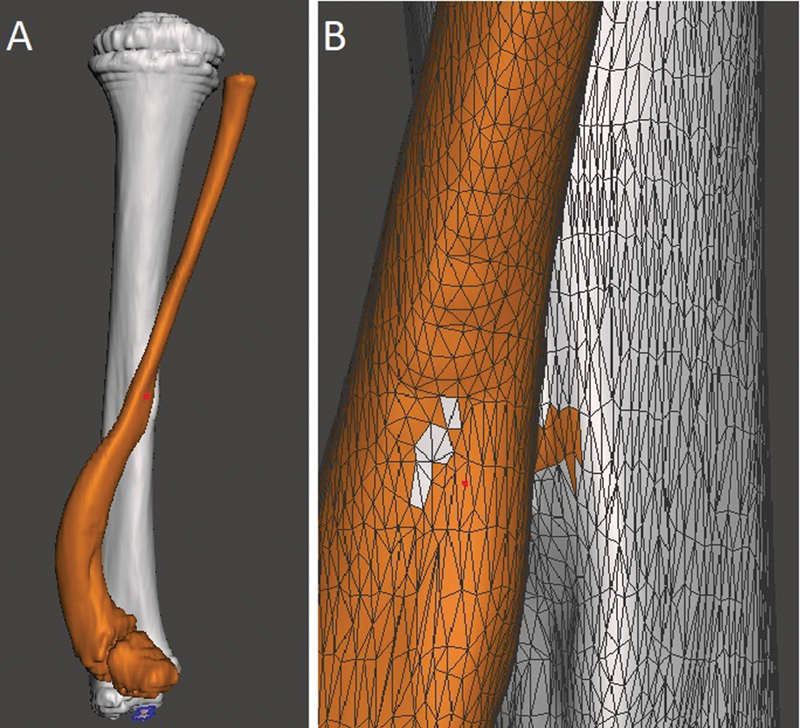
Selection of the bone for surgical planning. A shows the back view of the model and B shows an approximate view with the mesh to facilitate the correction of small selected defects. Image obtained by the authors using Meshmixer software (Autodesk Inc., San Rafael, CA, United States).

Now, select the bone for correction. This study used a fibula with a “serpentine” deformity. In the left sidebar, click on “Select” to open a navigation window with different selection methods. We find it easier to keep it in “Brush mode - SphereDisc Brush” and adjust the cursor size in “Size” as needed, ideally the smallest diameter of the bone to facilitate selection. When clicking the left button with this tool, you will notice the selection of the cursor area, marked by orange. The bone is marked along its entire length. If an erroneous area is marked, undo the last selection (Ctrl + Z) or deselect it with a Shift + left click. By pressing ''W'' and zooming in on the model, you get a better view for deformity correction during selection with a mesh view of the surface ( Fig. 1 ).

After the complete selection of the bone, instruct the software to recognize two independent objects, in this case, the tibia and fibula. Therefore, select the “Edit” option in the navigation window followed by “Separate”. In the “Object Browser”, you will see two options, one for each bone, and you can choose which structure to work with through this window or by clicking on the desired bone.

Changing the color makes it easier to see the two objects separately. When selecting the bone you want to change color, click on “Shaders” in the side menu and drag the chosen color to the object. You can also modify the name of each object in “Object Browser” by double-left-clicking on the name.

**Fig. 2:**
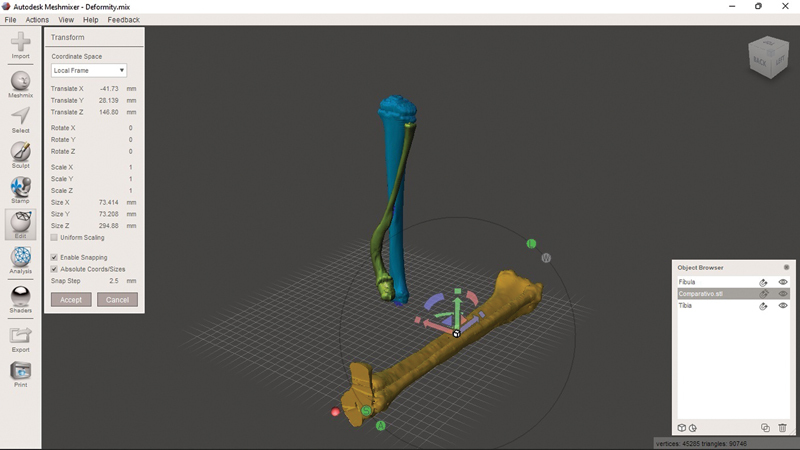
Three objects in the current model. Two objects present a deformity: the tibia (in blue) and the fibula (green). The unaffected tibia and fibula are in yellow. You can change the control object using the ''Transform'' menu and dragging the arrows (red/green/blue) to move it in the plane. Clicking on the half-moons (red/green/blue) allows you to rotate the axis. Clicking on the box (a common point of origin of the three arrows) resizes the object to fit the size of the affected bone. Image obtained by the authors using Meshmixer software (Autodesk Inc., San Rafael, CA, United States).

To compare the deformed bone with the unaffected one, add a new object. To do so, select “Import'” in the side menu, then the “Append” option in the pop-up menu, and navigate to the comparison SLT file to generate the object in the model plane. Apply a “Shader” to differentiate the object's color as previously described and, with it selected in the browser, click on “Edit” in the side menu. In the navigation window that appears, the “Transform” option allows you to change the size, movement, and rotation (with a goniometer) of the object in the plane ( Fig. 2 ).

**Fig. 3:**
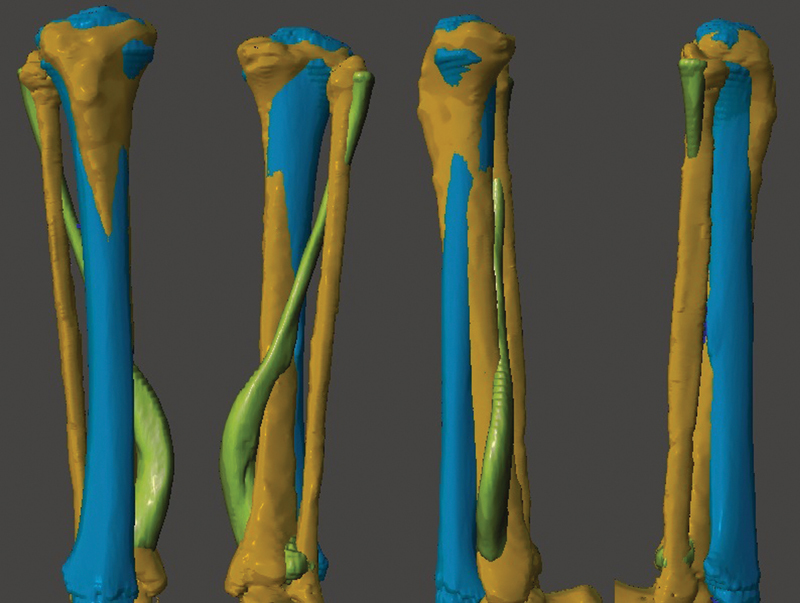
Superimposition of the three objects. From left to right, the figure shows the front, back, and side views of the model. Image obtained by the authors using Meshmixer software (Autodesk Inc., San Rafael, CA, United States).

Superimpose the control object over the deformed one using the arrows, half-moons, and resizing as described in Fig. 2 . The rotation is corrected by clicking “TOP” on the cube (top right corner) and aligning the tibial plateaus and the heads of the fibulae. In the end, you will have the model with the three objects superimposed, allowing an assessment of the deformity in multiple planes ( Fig. 3 ). You can hide or view objects independently by clicking on “Hide/Show Object” in “Object Browser”, represented by an eye.

**Fig. 4:**
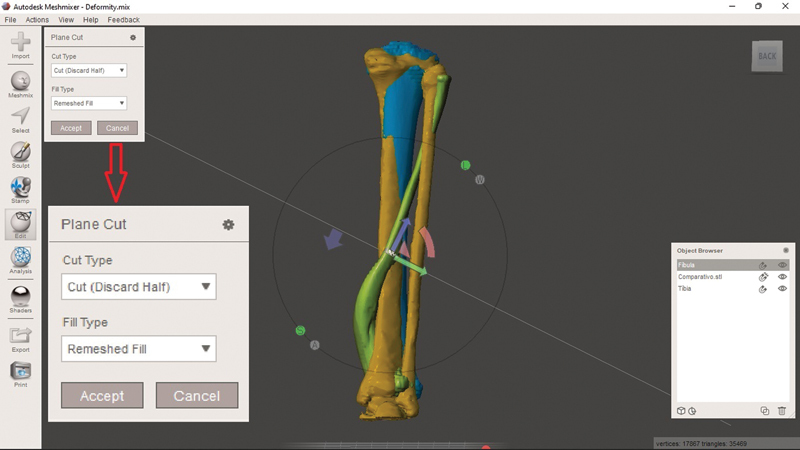
Sectioning tool, i.e., “Plane Cut”. You can modify the height and direction of the section by using the arrows and half-moons or when simulating the section (click and hold the left button at the starting point and drag the mouse to the endpoint). Note that the tool's navigation menu (enlarged in red arrow) is “Cut (Discard Half)” by default. This option discards part of the selected object. As such, select “Slice (Keep Both)”. Image obtained by the authors using Meshmixer software (Autodesk Inc., San Rafael, CA, United States).

With the objects superimposed, planning begins with sections (simulating the osteotomy) to approximate the deformity with the control comparative object. Select the bone you want to work with to section it alone. Select “Edit” in the sidebar and, in the navigation window that appears, click on “Plane Cut”. This generates a plan in the software interface that can be modified according to the desired sections using the arrows (as specified in Fig. 2 ) or simulate the section by dragging the cursor ( Fig. 4 ).

**Fig. 5:**
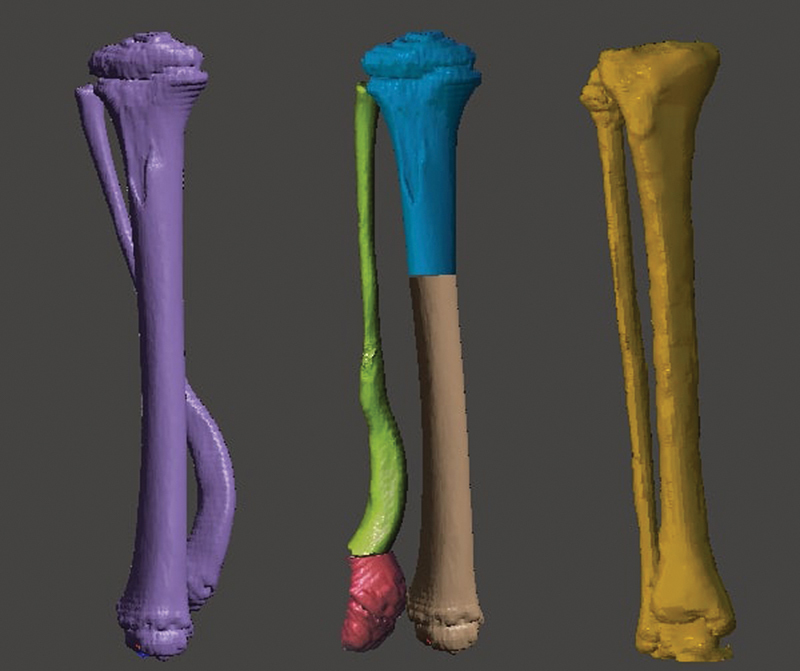
Comparison of the three different objects. From left to right: Deformity before correction, corrected deformity, and control object. Image obtained by the authors using Meshmixer software (Autodesk Inc., San Rafael, CA, United States).

When deciding the direction, pay attention to modifying the section type (“Cut Type”). The “Cut (Discard Half)” option will make the software discard half of the object after sectioning. Therefore, the desired option for planning osteotomies while maintaining the fragments is “Slice (Keep Both)”. Click on “Accept” and, in the opened navigation window, select “Separate Shells”, so that the software recognizes that the section generated two independent objects (note their appearance in the “Object Browser”). Each object generated by the section can now undergo editing with the “Transform” option, as previously described. After performing all the desired sections and modeling, compare them with the preoperative image and the control object ( Fig. 5 ).

## Final Comments

To date, software use for surgical planning is not widespread because of the apparently long learning curve or the requirement for software and hardware with a low cost-benefit ratio. 2 An easily accessible tool with a free license and an intuitive interface increases the efficiency of the processes to define a therapeutic plan and better understand the patient's deformity. 1 The software allows for broader modifications per the learning curve. Table 1 shows some examples of these functions. With no state-of-the-art 3D printers or specific computers, this technology allows a deeper study of the case and its particularities, such as direction, planes, and dimensions of the deformities. It also facilitates choosing osteotomy points and directions, mobilizing fragments, and selecting implants. It is believed that planning considering these topics reduces surgical time, resulting in a lower rate of postoperative complications and better functional outcomes due to an improved understanding of the deformity and the ways to correct it. Associating planning via software with 3D printing can further enhance the therapeutic plan definition.

**Table 1:** Some tools from the Meshmixer software (Autodesk Inc., San Rafael, CA, United States) to help planning, their effects, and practical examples

Tool	Effect	Use
Duplicate	Duplicate the selected object	To compare pre- and post-modeling; to perform multiple planning using the same object.
Mirror	Mirror the selected object	To mirror the patient's contralateral model for superimposition in cases of unilateral deformities.
Sculpt	Remove surfaces	To excise bony prominences for surgical planning. Example: benign bone tumors (osteochondromas).
Create Pivot	Create a pivot to change the axis of rotation on the selected object	Prosthetic planning and the joint assessment. Example: Evaluation of the mobility of the glenohumeral joint with a pivot in the center of rotation.
Units/dimensions	Estimate the distance between two points based on the scale of the generated model	Osteotomy height and fragment translation planning.
