# Supplementary material for: Methodology for Preoperative Planning of Bone Deformities Using Three-dimensional Modeling Software
Source: Rev Bras Ortop (Sao Paulo). 2024 Mar 21;59(1):e130–5. doi: 10.1055/s-0044-1779700 (PMC10957277; doi:10.1055/s-0044-1779700)
Supplement: Related texts

# Research article: Metodologia para planejamento pré-operatório de deformidades ósseas utilizando software de modelagem tridimensional

**Keywords:** impressão tridimensional, ortopedia, cuidados pré-operatórios

## Abstract

Observa-se o avanço da tecnologia de prototipagem rápida, conhecida como impressão tridimensional (3D) e seu uso na área médica. Existem estudos a respeito do tratamento de deformidades ósseas graves com impressão 3D, os quais mostram benefícios no resultado pós-operatório às custas do uso da tecnologia em questão. Ainda assim, nota-se a escassez quando o assunto é disponibilizar ao cirurgião orientações para planejamento pré-operatório. O objetivo desta nota técnica é descrever um passo-a-passo prático para auxiliar cirurgiões a utilizarem a tecnologia como ferramenta para otimizar o plano terapêutico, dispondo de um programa de licença gratuita e de interface intuitiva. Este é um estudo que visa a organização do processo de modelagem 3D, no qual foi utilizado um exame de tomografia computadorizada (TC) pré-operatória. Com esta tecnologia, é possível uma compreensão mais profunda do caso e suas particularidades como direção, planos e dimensões das deformidades. Acredita-se que um planejamento que leve em consideração tais tópicos gera redução do tempo cirúrgico e melhores resultados funcionais devido ao entendimento da deformidade e maneiras de correção. Associar o planejamento via *software* com a impressão 3D pode potencializar ainda mais na elaboração do método terapêutico.

## Introdução

Observa-se o avanço da prototipagem rápida, também conhecida como impressão tridimensional (3D) e seu uso na área médica.1 A tecnologia vem sendo aprimorada e aplicada de diversas formas e, na área ortopédica, tem sido utilizada para educação, melhor compreensão de doenças ou deformidades, personalização de implantes ou órteses sob medida e, também, para planejamento pré-operatório de procedimentos cirúrgicos. 1 2 3 4

Existem estudos envolvendo o tratamento de deformidades ósseas graves com impressão 3D, os quais mostram benefícios no resultado pós-operatório às custas do uso da tecnologia em questão. 5 Ainda assim, na literatura, nota-se a escassez quando o assunto é disponibilizar ao cirurgião orientações para um planejamento pré-operatório utilizando a prototipagem rápida.

Visto que existem múltiplos programas para modelagem 3D 6 7 com variadas curvas de aprendizagem e que, à primeira vista, podem parecer complexos, o objetivo da presente nota técnica é descrever um passo-a-passo prático para auxiliar cirurgiões ortopédicos a utilizarem a tecnologia como ferramenta para otimizar o plano terapêutico, dispondo de um programa de licença gratuita e de interface intuitiva.

## Descrição Dos Materiais e Técnica

O projeto de pesquisa foi aprovado pelo Comitê de Ética em Pesquisa do hospital (CAAE: 48296321.8.0000.0097).

Este é um estudo que visa a organização do processo de modelagem 3D por *software* , no qual foi utilizado o exame de tomografia computadorizada (TC) pré-operatória da perna de um paciente portador de Síndrome de Hajdu-Cheney com deformidade de fíbula "em serpentina". O exame foi realizado no aparelho de TC *multislice* do hospital, GE Healthcare Revolution, 64 canais (GE Healthcare, Chicago, IL, Estados Unidos).

Para a reconstrução das imagens, recorreu-se ao *software InVesalius* (Centro de Tecnologia da Informação Renato Archer, Brasil) versão 3.1.1; e o *software* utilizado para a modelagem e edição foi o *Autodesk Meshmixer* ( *Autodesk Inc* ., San Rafael, CA, Estados Unidos) versão 3.5.474.

## Reconstrução da Imagem

Para o planejamento no *Meshmixer* , são necessários dois arquivos no formato *stereolithography* (STL, na sigla em inglês), sendo um do osso com a deformidade que está sendo estudada e outro com o mesmo osso sem deformidade, o qual servirá de comparação.

Para isso, primeiramente, realiza-se o *download* da imagem da TC do paciente diretamente do sistema de visualização disponível no serviço de imagens/hospital. O arquivo deve ser em formato *digital imaging and communications in medicine* (DICOM) para que seja importado ao software *InVesalius* . A seleção de área de interesse para a conversão em STL são realizadas conforme descrito por Pontes et al *.* 8

O arquivo STL de comparação pode ser obtido com a TC contralateral em casos de deformidades unilaterais ou de outro paciente de faixa etária semelhante.

## Planejamento Cirúrgico: Edição e Modelagem 3D

Inicializar o *software Meshmixer* , selecionar a opção *‘’import''* e escolher o arquivo SLT da deformidade. A imagem 3D será carregada. Inicialmente, é importante se familiarizar com alguns comandos que serão necessários para prosseguir com a modelagem.

Com o clique direito do *mouse* , permite-se a rotação do modelo. O botão de rolagem do *mouse* permite aproximar ou afastar do modelo e o clique com o botão de rolagem permite a navegação nos planos horizontal e vertical.

Nota-se duas barras principais na interface: a superior e a lateral esquerda. Nesta, temos as principais ferramentas para edição: *‘’Import''* , *‘’Meshmix''* , *‘'Select’'* , *‘'Sculpt’* ', *‘'Stamp’'* , *‘’Edit''* , *‘’Analysis'* ', *‘'Shaders’'* , *‘’Export''* e *‘’Print''* . Para o planejamento cirúrgico com osteotomias, inicialmente, as principais funções são *‘’Edit'* ' que permite a realização dos cortes e a mobilização de fragmentos e *‘'Shaders’* ' que permite mudança nas cores, facilitando a visualização.

Na barra superior do programa, nota-se os comandos *‘’File''* , *‘’Action''* , *‘’View''* , *''Help'* ' e *‘’Feedback''* . Ao clicar em " *View"* , marca-se a opção *‘'Show objects browser''* , pois isso facilitará a edição nos passos seguintes. Ao clicar pela primeira vez, nota-se um navegador de objetos ( *‘’Object Brow* ser'' *)* , atualmente, com um único objeto.

Agora, deve-se selecionar o osso a ser corrigido. Neste artigo, utilizamos a fíbula com deformidade "em serpentina". Na barra lateral esquerda, clica-se em *‘'Select’'* . Será aberta uma janela de navegação, com diversas formas de seleção. Encontramos maior facilidade mantendo no *‘’Brush mode - SphereDisc Brush''* e ajustando o tamanho do cursor em *‘'Size’'* conforme necessidade, idealmente o tamanho do menor diâmetro do osso para facilitar a seleção. Ao clicar com o botão esquerdo com essa ferramenta, nota-se a seleção da área do cursor, marcada pela cor alaranjada. Marca-se o osso em toda a sua extensão. Caso seja marcada uma área errônea, podemos desfazer a última seleção ( *Ctrl* *+* *Z* ) ou então desmarcar com *Shift* + clique esquerdo. Ao pressionarmos ‘’W'' e aproximarmos ao modelo, conseguimos uma visualização melhor para correção de falhas durante a seleção com a visualização em malha da superfície ( Fig. 1 ).

**Fig. 1:** Seleção do osso em que deseja realizar o planejamento cirúrgico. Vista posterior do modelo em A, e vista aproximada com malha em B, facilitando correção de pequenos defeitos de seleção. Imagem obtida pelos autores no *software Meshmixer* ( *Autodesk Inc.* , San Rafael, CA, Estados Unidos).

Após a seleção completa do osso, deve-se orientar o programa a reconhecer dois objetos independentes, no caso, tíbia e fíbula. Portanto, seleciona-se a opção ‘’ *Edit* '' na janela de navegação seguido de ‘' *Separate* ’'. No navegador de objetos ( *Object Browser* ), vemos agora duas opções, uma para cada osso, e pode-se escolher com qual estrutura trabalhar por esta janela, ou ao clicar no osso desejado.

A alteração da cor facilita a visualização dos dois objetos separadamente. Ao selecionar o osso que deseja alterar cor, clicar em ‘' *Shaders* ’' no menu lateral e arrastar a cor escolhida ao objeto. Pode-se também modificar o nome de cada objeto no ‘’ *Object Browser''* ao duplo-clique esquerdo no nome.

Para comparação do osso deformado com o osso de anatomia fisiológica, deve-se adicionar um novo objeto: selecione ‘’ *Import* '' no menu lateral, seguido da opção ‘’ *Append* '' no *pop-up* e navegue ao arquivo SLT do comparativo, gerando o objeto no plano do modelo. Aplica-se um " *Shader* " para diferenciar a cor do objeto conforme descrito previamente e, com ele selecionado em nosso navegador, escolher ‘’ *Edit* '' no menu lateral. Na janela de navegação que surge, a opção ‘’ *Transform* '' permitirá a alteração do tamanho, movimentação e rotação (com goniômetro) do objeto no plano ( Fig. 2 ).

**Fig. 2:** Três objetos em modelo atual, sendo dois de deformidade: tíbia (azul) e fíbula (verde) e um controle, tíbia e fíbula, em amarelos. O objeto controle pode ser alterado pelo menu ‘‘ *Transform* ’': ao arrastar as setas (vermelha/verde/azul), movimenta-se o mesmo no plano. Clicar nas meias-luas (vermelha/verde/azul) permite rotacionar em seu próprio eixo. Clicar na caixa (ponto de origem em comum das três setas) redimensiona o objeto para que ele se adeque ao tamanho dos objetos com a deformidade. Imagem obtida pelos autores no *software Meshmixer* ( *Autodesk Inc.,* San Rafael, CA, Estados Unidos).

Deve-se sobrepor o objeto normal ao objeto com deformidade usando as setas, meias-luas e redimensionamento conforme descrito na Fig. 2 . A rotação é corrigida ao clicar em ‘’ *TOP* '' no cubo (canto superior direito) e alinhar os planaltos tibiais e as cabeças das fíbulas. Ao final, teremos o modelo com os 3 objetos sobrepostos, permitindo uma avaliação da deformidade em múltiplos planos ( Fig. 3 ). Pode-se ocultar ou visualizar objetos de forma independente ao clicar em *‘’Hide/Show Object''* no *‘’Object Browser''* , representado pelo desenho de um olho.

**Fig. 3:** Sobreposição dos três objetos. Da esquerda para a direita, vistas anterior, posterior e laterais do modelo. Imagem obtida pelos autores no *software Meshmixer* ( *Autodesk Inc.* , San Rafael, CA, Estados Unidos).

Com a sobreposição dos objetos, inicia-se o planejamento com cortes (simulando a osteotomia) visando a aproximação da deformidade com o objeto comparativo normal. Deve-se selecionar o osso com o qual deseja trabalhar para que os cortes afetem apenas a ele. Seleciona-se *‘’Edit''* na barra lateral e, na janela de navegação que aparece, clicar em *‘’Plane Cut''* . Isso gera um plano na interface do programa que pode ser modificado de acordo com o corte desejado pelas setas (de uso igual ao especificado na Fig. 2 ) ou simulando o corte ao arrastar o cursor ( Fig. 4 ).

**Fig. 4:** Ferramenta de corte: ‘’ *Plane Cut''* . A altura e direção do corte podem ser modificadas ao utilizar as setas e meias-luas ou ao simular o corte (clicar e segurar com o botão esquerdo no ponto de início e arrastar o *mouse* até o ponto final). Nota-se no menu de navegação da ferramenta (ampliado em seta vermelha) a sua padronização por ‘’ *Cut (Discard Half)''* . Caso mantenha esta opção, o corte provocará o descarte de parte do objeto selecionado. Por isso, deve-se selecionar ‘' *Slice (Keep Both)’'.* Imagem obtida pelos autores no *software Meshmixer* ( *Autodesk Inc.* , San Rafael, CA, Estados Unidos).

Ao decidir a direção, deve-se atentar para modificar o tipo de corte ( *‘’Cut type'')* . A opção *‘’Cut (Discard Half)”* fará com que o *software* descarte metade do objeto após o corte. Portanto, a opção desejada para planejamento de osteotomias mantendo os fragmentos é *‘'Slice (Keep Both)’'* . Clicar em *‘’Accept''* e, na janela de navegação aberta, selecionar *‘'Separate Shells’'* , para que o *software* reconheça que o corte gerou dois objetos independentes (notar o aparecimento no *‘’Object Browser'')* . Cada objeto gerado pelo corte, agora, pode ser editado com a opção *‘’Transform''* , conforme uso descrito previamente. Após realizar todos os cortes e modelagem desejados, pode-se comparar com o pré-operatório e com objeto controle ( Fig. 5 ).

**Fig. 5:** Comparativo entre três objetos diferentes. Da esquerda para a direita: Deformidade antes da correção, deformidade corrigida, objeto controle. Imagem obtida pelos autores no *software Meshmixer* ( *Autodesk Inc.* , San Rafael, CA, Estados Unidos).

## Comentários Finais

Até a presente data, o uso de *softwares* para planejamento cirúrgico não é amplamente difundido devido à impressão de uma longa curva de aprendizagem ou requisição de *softwares* e *hardwares* que não justifiquem o custo-benefício. 2 Com uma ferramenta de fácil acesso, de licença gratuita e interface intuitiva, os processos de definição de um plano terapêutico e melhor entendimento da deformidade do paciente são atingidos de forma mais eficaz. 1 O *software* em questão permite modificações mais amplas, associadas à sua curva de aprendizagem. Alguns exemplos destas funções são citados na Tabela 1 . Sem necessidade de impressoras 3D de última geração ou computadores específicos, com esta tecnologia, é possível um estudo mais profundo do caso e suas particularidades como direção, planos e dimensões das deformidades. Consegue-se, também, facilitar na escolha de pontos e direções de osteotomias, mobilizar fragmentos e escolher implantes. Acredita-se que um planejamento que leve em consideração tais tópicos gera redução do tempo cirúrgico, o que está associado a um menor índice de complicações pós-operatórias e a melhores resultados funcionais devido ao entendimento da deformidade e maneiras de correção. Associar o planejamento via *software* com a impressão 3D pode potencializar ainda mais na definição do plano terapêutico.

**Tabela 1:** Algumas ferramentas do *software Meshmixer* ( *Autodesk Inc.* , San Rafael, CA, Estados Unidos) que podem auxiliar no manejo do planejamento, seus efeitos e exemplos para uso

Ferramenta	Efeito	Uso
*Duplicate*	Duplicar o objeto selecionado	Comparar pré e pós modelagem; múltiplos planejamentos com o mesmo objeto.
*Mirror*	Espelhar o objeto selecionado	Permitir o espelhamento do modelo contralateral do paciente para sobreposição em casos de deformidades unilaterais.
*Sculpt*	Permitir a remoção de superfícies	Excisar proeminências ósseas em planejamento cirúrgico. Exemplo: tumores ósseos benignos (osteocondromas).
*Create Pivot*	Gerar um pivô para alterar o eixo de rotação no objeto selecionado.	Permitir o planejamento com próteses e estudo de articulações. Exemplo: Estudo da mobilidade da articulação glenoumeral com pivô no centro de rotação.
*Units/dimensions*	Estimar a distância entre dois pontos com base na escala do modelo gerado	Auxiliar no planejamento para altura de osteotomias e translação de fragmentos.
